# Incidence and risk factors of cataract following pediatric pars plana vitrectomy

**DOI:** 10.1186/s40942-025-00718-w

**Published:** 2025-08-20

**Authors:** Antoine Bourgeois, Thibaut Chapron, Ismael Chehaibou, Florence Metge, Youssef Abdelmassih, Georges Caputo

**Affiliations:** 1 Pediatric Ophthalmology Department, Rothschild Foundation Hospital, 29 rue Manin, Paris, 75019 France; 2 Sorbonne Universités, Paris, France; 3 Obstetrical Perinatal and Pediatric Epidemiology Research Team, EPOPé, Université Paris Cité, CRESS, INSERM, INRAE, Paris, F-75004 France

## Abstract

**Purpose:**

To evaluate the incidence and risk factors for cataract following pediatric pars plana vitrectomy (PPV).

**Setting:**

Tertiary referral center.

**Design:**

Retrospective consecutive case series.

**Methods:**

We included 242 eyes of 218 patients (< 18 years) that underwent lens-sparring PPV with a minimum follow-up of 6 months. Information regarding demographic and ophthalmic features, surgical history and procedures were gathered and analyzed. Eyes were evaluated for the development of cataract requiring surgery.

**Results:**

Mean age at surgery was 6.8 ± 5.0 years and mean follow-up was 31.9 ± 37.5 months. After a mean interval of 17.0 ± 22.0 months, 80 eyes (33.1%) required lensectomy with posterior subcapsular cataract being the most common cataract type (76.3%). Overall, the risk of developing cataract after PPV increased from 21% at 1 year to 47% at 5 years The factor associated with the development of postoperative cataract were the need for multiple surgeries (58.8% vs. 13.6%, *p* < 0.001), the type of tamponade used *p* < 0.001), older age at surgery (101.9 ± 53.1 months vs. 71.0 ± 60.8 months, *p* < 0.001), and retinal detachment (78.8% vs. 19.1%, *p* < 0.001). Multivariate analysis including the aforementioned variables identified the need for multiple surgeries [OR: 2.7 (CI: 1.2–6.2), *p* = 0.02)] as a risk factor for the development post-PPV cataract while the use of air or no tamponade as protective (*p* = 0.001).

**Conclusions:**

Post-PPV cataract is a common complication occurring in about one-third of children. Risk factors include silicone oil tamponade, gas tamponade and multiple surgeries. Follow-up should be started early and continued for an extended duration after PPV especially in young children at risk of developing amblyopia.

## What was known

- Lens sparing pars plana vitrecomy is being performed is becoming more prevalent.
- Cataract following pars plana vitrectomy seems to occur less frequently than in adults.

## What this paper adds

- Of 242 eyes undergoing pediatric pars plana vitrectomy, 33.1% developed cataract.
- The 5-year cataract incidence was 47% overall; 15% with no or only air, 31% with gas and 86% with silicone oil.
- Key risk factors included tamponade (silicone oil and gas) and multiple surgeries.

## Background

Pars plana vitrectomy (PPV) results in the progression of pre-existing nuclear sclerosis and the development of a new cataract [1]. The cataract formation results in decreased visual acuity within 1 to 2 years after PPV and the need to perform cataract surgery [2, 3]. Several factors have been associated with the development of post-PPV cataract such a the age at surgery, diabetes, the type of infusion fluid, the need and the type of tamponade (air, gas or silicone oil), and the duration of surgery and exposure to light source [4–10].

In the adult population, cataract progression occurred in up to 100% of patients with almost 60% of patients undergoing cataract surgery in the 2 years following PPV [2, 11, 12]. Lower frequency of post-PPV cataract was reported in young population (< 50 years) ranging from 7 to 60% [11, 13–15]. Thompson reported a significantly lower nuclear sclerosis progression in patients younger than 50 years compared to older patients [3]. Little is known about the risk of post-PPV cataract in pediatric population.

Pediatric lens sparing PPV is an increasingly common practice that has already proven its worth in various pediatric retinal pathologies, and accounts for about 2% of total PPV performed [16]. Preserving the crystalline lens during PPV avoids aphakia, facilitates visual rehabilitation, and limits the risk of postoperative amblyopia [17, 18]. The rate of post-PPV cataract varies from 5 to 34% in studies with a limited number of patients, and with retinopathy of prematurity (ROP) representing the main etiology [5, 18–22]. The scientific literature on the incidence and specific risk factors for post-PPV cataract in pediatric population remains limited. Understanding these aspects is essential to improve the management and follow-up of these patients.

The aim of the present retrospective study was to estimate the incidence of and to determine the risk factors for cataract development following PPV in a pediatric population.

## Methods

In this retrospective consecutive case series, we included all patients under the age of 18 that underwent lens sparing PPV at the Rothschild Foundation Hospital, a tertiary referral center, between January 2002 and December 2023. We excluded patients with a postoperative follow-up of less than 6 months, a preexisting cataract, and patients that underwent initial lensectomy. The research was approved by the Rothschild Foundation Hospital review board – IRB00012801- under the study number CE_20231128_15_YAH and adhered to the tenets of the declaration of Helsinki.

Data was retrospectively extracted from a medical record software (*Softalmo*,* Corilus*,* France*) and included age at initial surgery, gender, indication for PPV, history of trauma, total number of intraocular surgeries, surgical details, the development of cataract, cataract type, and interval between PPV and lensectomy. Data regarding surgery procedures included: instrument-crystalline lens touch, type and number of tamponades, postoperative positioning, and duration of silicone tamponade. Bilateral surgeries were performed sequentially. No cataract grading system was used. The management of persistent fetal vasculature was discussed in previous papers [23, 24]. Lensectomy was performed in case of any opacity affecting the visual axis or hindering fundus examination and when the posterior subcapsular cataract (PSC) was located centrally as established by previous studies [21, 22]. Following lens sparing PPV, patients were examined one week, one month and at least every 6 months thereafter to evaluate the presence of cataract and other postoperative complications such as ocular hypertension and recurrence of retinal detachment (RD).

### Statistical analysis

Statistical analysis was done using the IBM SPSS software (version 25.0, Chicago, IL). Continuous variables were presented as mean ± standard deviation (SD), and were compared using the dependent or independent Student’s t-test. Categorical variables were reported as percentages (%) and compared using chi-square test. Multivariate analysis was then used to control for confounding factors and to calculate the odds ratio for the development of cataract. Time-related occurrence of cataract necessitating surgery was analyzed using the Kaplan-Meier estimator. All tests were conducted with a two-tailed formulation. A p-value < 0.05 was considered statistically significant.

## Results

### Baseline characteristics

A total of 218 patients (242 eyes), with a mean age at surgery of 6.8 ± 5.0 years (range: 1 month- 18 years), were included. Bilateral surgery was performed in 24 patients (10%), and multiple surgeries were needed in 69 eyes (28.5%) with a mean number of surgeries of 1.3 ± 0.59. The main etiologies for PPV were: RD in 94 eyes (38.8%), epiretinal membrane peeling in 38 eyes (15.7%), ROP in 28 eyes (12.4%), and vitreous hemorrhage in 27 eyes (11.2%). The ERM was idiopathic in 14 patients and secondary in the remaining cases including familial exudative vitreoretinopathy in 12 eyes and combined hamartoma in 4 eyes. Eyes with ROP were stages 4 A and 4B (27 eyes) and one eye with stage 3. At the initial surgery, no postoperative tamponade was needed in 98 eyes (40.5%), silicone oil in 78 eyes (32.2%), gas in 41 eyes (16.9%), air in 17 eyes (7.0%), and tamponade was not reported for 8 eyes (3.3%). Multiple tamponades were only needed in 6 eyes (2.5%). The mean postoperative follow-up was 31.9 ± 37.5 months (range: 6-240). Cataract was reported in 80 eyes (33.1%) after an average 17.0 ± 22.0 months following PPV and the PSC was the most commonly reported cataract type in 61 out of 80 eyes (76.3%) with a mean interval between the development of cataract and the surgical management of 7.3 ± 9.3 months (range 0 months- 60 months). Per operative lens touch occurred in only 4 eyes (5%) where lens ablation was performed during the initial surgery (Table 1).

**Table 1:** Baseline characteristics on included patients. CS: centistoke; ERM: epiretinal membrane; MH: macular hole; PFV: persistent fetal vasculature; PPV: Pars plana vitrectomy; RD: retinal detachment; ROP: retinopathy of prematurity; SD: standard deviation; VH: vitreous hemorrhage

	242 eyes (218 patients)
Age in years (mean ± SD)	6.8 ± 5.0 years
Bilateral surgery (%)	24 patients (10%)
Multiple surgery (%)	69 eyes (28.5%)
Mean number of surgeries (mean ± SD)	1.3 ± 0.59
*PPV etiology (%)*	
RD ROP PFV ERM MH Optic pit VH Other	94 (38.8%) 30 (12.4%) 19 (7.9%) 38 (15.7%) 13 (5.4%) 11 (4.5%) 27 (11.2%) 10 (4.1%)
History of trauma (%)	32 (13.2%)
*Initial tamponade (%)*	
None Air SF6 C2F6 C3F8 Silicone oil (1000cs) Missing	98 (40.5%) 17 (7.0%) 12 (5.0%) 25 (10.3%) 4 (1.7%) 78 (32.2%) 8 (3.3%)
Mean duration of silicone oil tamponade in months (mean ± SD)	7.2 ± 7.5
Multiple tamponade (%)	6 eyes (2.5%)
Mean follow-up in months (mean ± SD)	31.9 ± 37.5 months
Postoperative cataract (%)	80 (33.1%)
Type of cataract (%)	
Posterior subcapsular Cortical Nuclear Lens touch	61 (76.3%) 9 (11.3%) 6 (7.5%) 4 (5.0%)
Mean interval for cataract development in month (mean ± SD)	17.0 ± 22.0

### Risk factors associated with the development of cataract

Eyes that developed cataract were more likely to have undergone multiple surgeries (58.8% vs. 13.6%, *p* < 0.001), and were operated at an older age (101.9 ± 53.1 months vs. 71.0 ± 60.8 months, *p* < 0.001). The etiology affected the development of cataract (*p* < 0.001) with RD being more likely to develop cataract compared to the remaining etiologies (67.0% vs. 11.5%, *p* < 0.001). Similarly, the type of tamponade used affected the risk of developing postoperative cataract: 9.6% with air or no tamponade, 17.1% with gas, and 78.2% with silicone oil (*p* < 0.001).

Multivariate analysis for the development of post-PPV cataract including the type of tamponade, the need for multiple surgeries, the age at surgery, and RD etiology was performed. Only the need for multiple surgeries [OR: 2.6 (CI: 1.1-6.0), *p* = 0.03)] was found to be associated with a higher risk of cataract development, while the use of air or no tamponade was protective against the development of postoperative cataract compared to gas [OR: 0.13 (CI: 0.04–0.44), *p* = 0.001)] and to silicone oil [OR: 0.09 (CI: 0.02–0.36), *p* = 0.02)] (Table 2).

**Table 2:** Effects of different variables on the development of cataract needing surgery with univariate and multivariate analysis. OR: odds ratio; PPV: Pars plana vitrectomy; SD: standard deviation. *P* < 0.05 is statistically significant

	Cataract (80 eyes)	No cataract (162 eyes)	*p*-value	OR	*p*-value
Age at surgery in months (mean ± SD)	101.9 ± 53.1	71.0 ± 60.8	< 0.001	1.0	0.98
Air or no tamponade (%)	11 (13.8%)	104 (64.2%)	< 0.001		
Gas (%)	7 (8.6%)	34 (20.9%)		0.13	0.001
Silicone oil (%)	61 (76.3%)	17 (10.5%)		0.09	0.001
Multiple PPV (%)	47 (58.8%)	22 (13.6%)	< 0.001	2.6	0.03
Retinal detachment (%)	63 (78.8%)	31 (19.1%)	< 0.001	2.1	0.22

### Kaplan-Meier survival curve

**Fig. 1:**
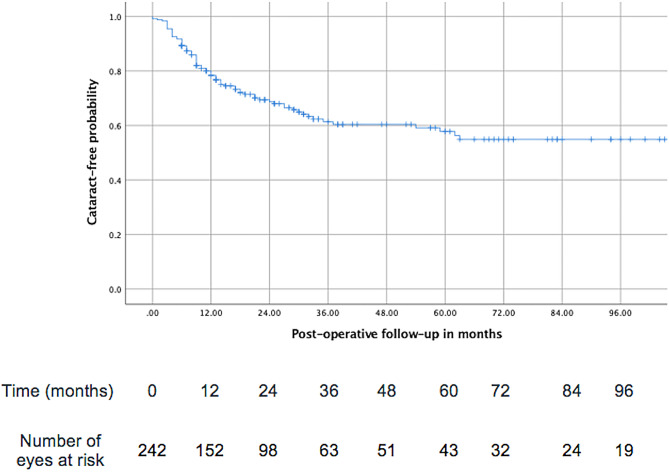
Kaplan-Meier curve showing the probability of needing cataract surgery over time after pars plana vitrectomy and table resuming the number of eyes at risk of receiving cataract surgery over time

**Fig. 2:**
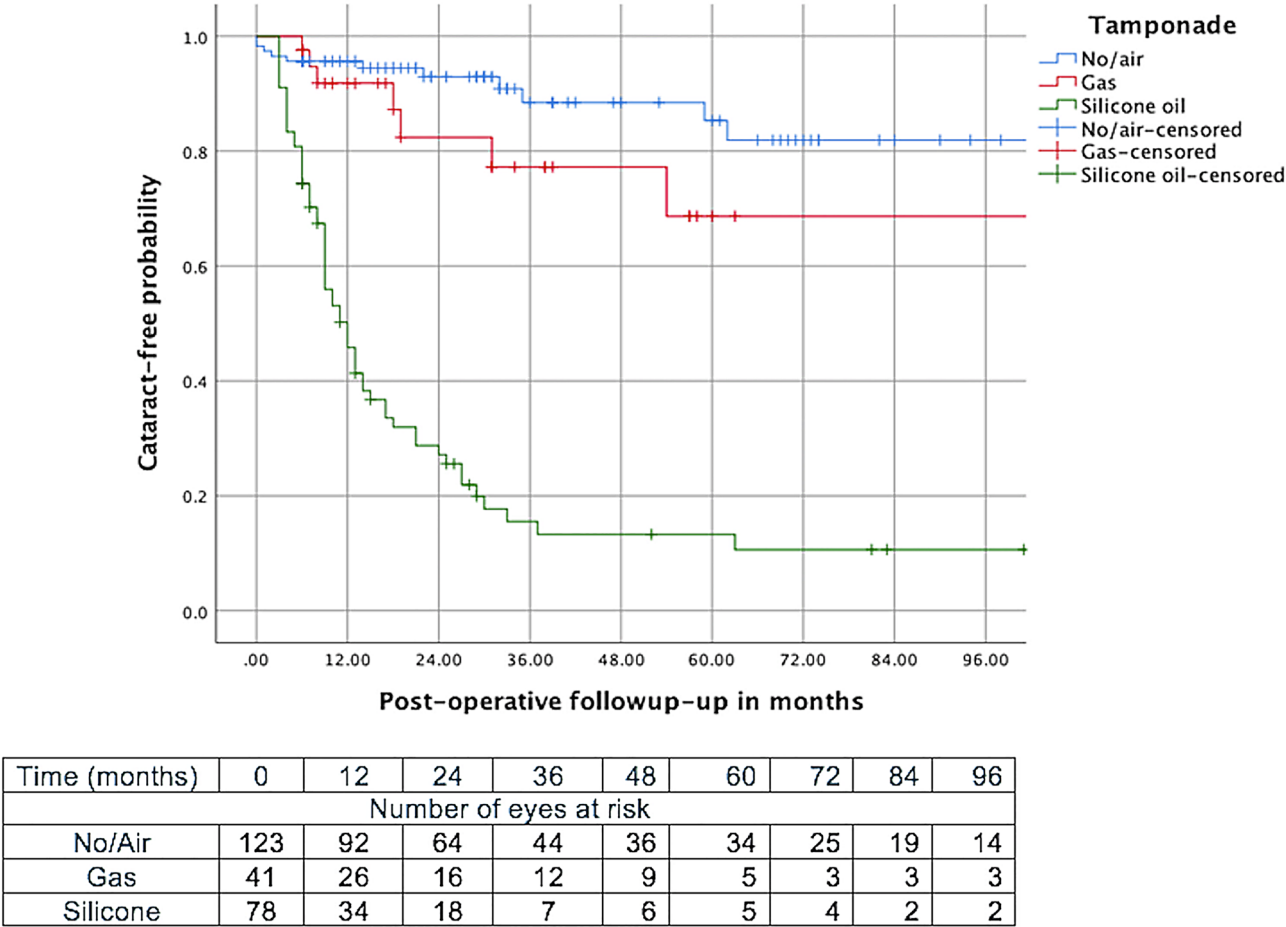
Kaplan-Meier survival curve showing the probability of developing a cataract after pars plana vitrectomy according to the use of silicone oil tamponade, gas, or air/no tamponade

A Kaplan-Meier survival curve of eyes remaining cataract-free over time is presented in Fig. 1. The cumulative risk of developing cataract after PPV increased from 21% at 1 year [Standard error (SE): 3%] to 47% at 5 years [SE:4%]. The incidence of cataract was significantly higher in eyes receiving silicone oil tamponade (*p* < 0.001). The cumulative risk for developing cataract increased from 50% at 1 year (SE: 6%) to 86% at 5 years (SE:5%) in the eyes receiving silicone oil, from 8% at 1 year (SE: 4%) to 31% at 5 years (SE:11%) in eyes receiving gas and from 4% at 1 year (SE: 2%) to 15% at 5 years (SE:5%) in eyes receiving no or only air tamponade (Fig. 2).

**Fig. 3:**
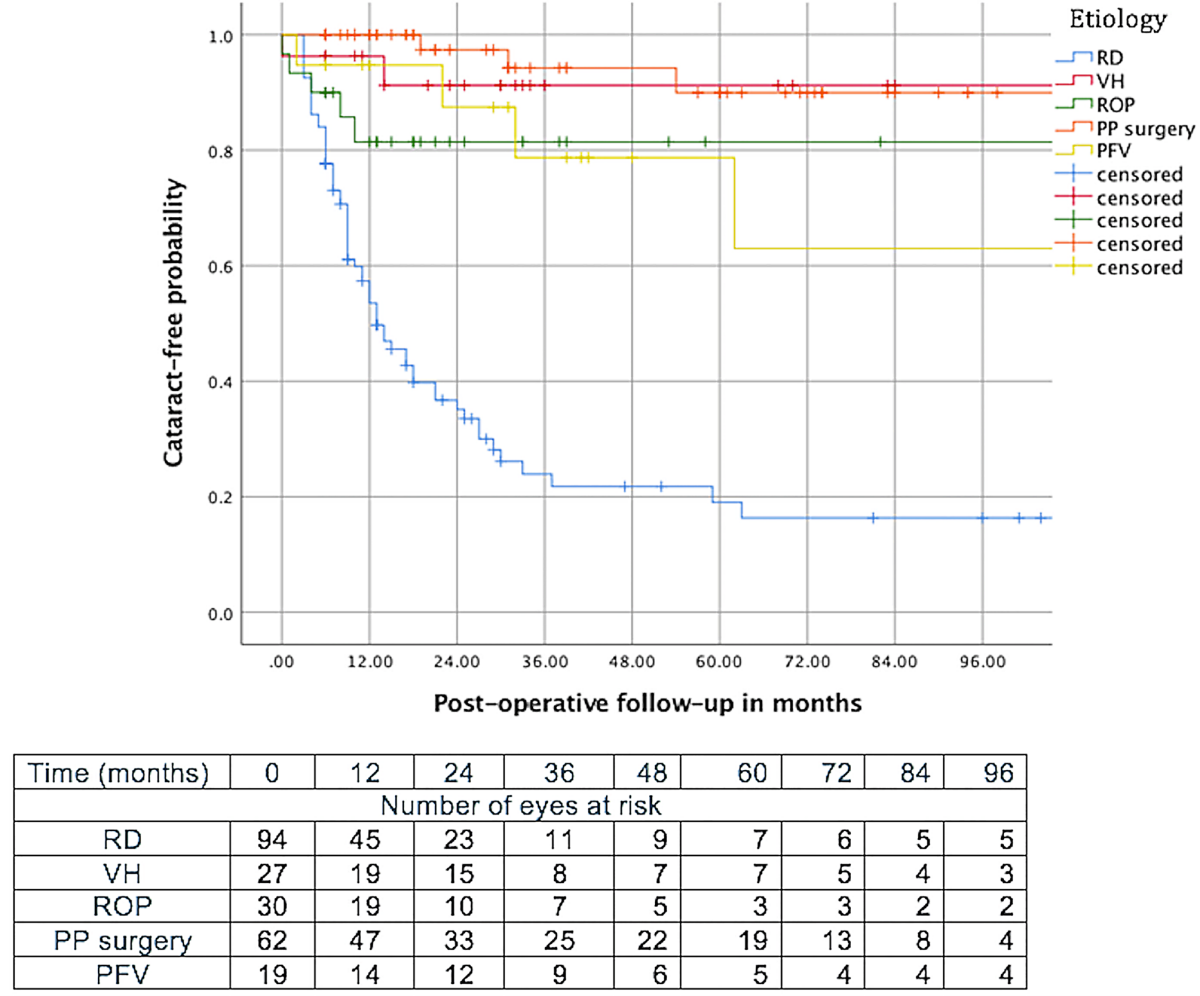
Kaplan-Meier survival curve showing the probability of developing a cataract after pars plana vitrectomy according to the initial etiology. PFV: Persistent fetal vasculature; PP: posterior pole; RD: Retinal detachment); ROP: retinopathy of prematurity; VH: Vitreous hemorrhage (VH)

Similarly, the etiology for PPV influenced the development of cataract (*p* < 0.001), with RD having the highest incidence, followed by PFV and ROP, and then by posterior pole surgery and vitreous hemorrhage with respective mean survival time of 52.8 months (SE: 11.2), 105.6 months (SE: 16.7), 109.2 months (SE: 10.6), 113.2 months (SE: 5.6), and 183.1months (SE:11.5) (Fig. 3).

## Discussion

In this retrospective cases series, we reviewed all patients under the age of 18 who underwent lens sparing PPV and assessed the development of postoperative cataract necessitating lensectomy. After an average follow-up of 31.9 ± 37.5 months, 80 eyes (33.1%) underwent lensectomy after a mean interval of 17.0 ± 22.0 months following PPV. The cumulative risk of lensectomy increased from 21% at 1 year to 47% at 5 years. Similar findings were reported by Fernandez et al., who observed that 61% of 44 eyes undergoing lens-sparing PPV developed some degree of cataract, with 34% requiring lensectomy during follow-up [5]. However, the rate of cataract formation in our study was lower compared to older populations, where up to 60% of patients required lensectomy within two years of PPV [2]. Jackson et al. reported that 52.1% of patients undergoing PPV for RD and 64.6% of patients undergoing PPV for macular hole required cataract surgery within one year [25, 26]. The lower cataract incidence in younger populations could be related to the crystalline lens in infants better tolerating the metabolic insult caused by PPV [27].

In our cohort, 6 eyes that underwent surgery for ROP needed lensectomy of whom 2 had a lens touch. The rate of eyes maintaining a clear lens at the final follow-up was 86% (24 out of 28 eyes). Lower rates of cataract formation were noted in studies focused exclusively on ROP cases, with Nudleman et al. reporting that only 5.9% of eyes required lensectomy due to lens opacity and Lakhanpal noting that 94.4% of eyes maintained clear lenses at the final follow-up [20, 22]. Our results were similar to Iwahashi et al. who found that nearly 10% of eyes required lensectomy because of lens opacity after lens sparing vitrectomy for ROP with a 10-year Kaplan-Meier of proportion of phakic eyes of 89% [19]. The lower cataract incidence in these studies may be attributed to the lesser need for tamponade during ROP surgeries, shorter follow-up durations, and different premenstrual age.

In our study, PSC was the most frequently observed type of cataract (76.3%). Similarly, Blodi et al. found that PSC was the most common type of cataract (57% of eyes) in a younger population (< 30 years) following PPV [15]. In contrast, nuclear sclerosis tends to be more prevalent in older populations [2, 3]. Younger patients are more likely to develop PSC following PPV, while older patients (> 50 years) are more prone to developing or experiencing progression of preexisting nuclear sclerotic cataract [1, 28].

Silicone oil tamponade and gas tamponade was more frequent in eyes that underwent lensectomy than no or air tamponade (*p* < 0.001) and increased the risk of cataract development by 11.1-fold (*p* = 0.001) and 7.7-fold (*p* = 0.001) respectively. Karel and Michaličková found that all eyes receiving silicone oil tamponade developed cataract [4]. Fernandez et al. corroborated our results, notably in multivariate analysis, showing that the use of both C3F8 gas and silicone oil significantly increased the risk of cataract development and the subsequent need for post-PPV lensectomy [5]. Similar findings were reported in adults by Antoun J et al., who observed an 80% rate of lens extraction following silicone oil tamponade in eyes that underwent PPV for RD [6]. Both silicone oil and gas tamponades have been associated with post-PPV cataracts, with gas tamponades contributing to transient or permanent lens feathering and silicone oil leading to subcapsular opacification [1, 29, 30].

Patients with RD were more likely to develop post-PPV cataract with univariate analysis but not with multivariate analysis. However, the need for multiple PPV surgeries increased the risk of post-PPV cataract by 2.7-fold in both univariate and multivariate analyses (*p* = 0.02). We believe that patients with RD are more likely to require multiple surgeries compared to other conditions, which increases the likelihood of using tamponades. Eyes requiring multiple PPV procedures often have more complex pathologies, such as proliferative vitreoretinopathy, and are exposed to a greater risk of post-PPV cataract formation due to inflammation, prolonged postoperative corticosteroid use, and increased oxidative stress on the lens [1, 15].

Kaplan-Meier survival curve analysis revealed three distinct groups with increasing risks of post-PPV cataracts: vitreous hemorrhage and posterior pole surgeries carried a mild risk; ROP and PFV had a moderate risk; and RD carried the highest risk. The relatively low risk in the first group may be attributed to the limited need for tamponades, shorter operative times, and less extensive PPV [31]. In the second group, opening the anterior hyaloid, performing retro-lenticular dissections and manipulations increase the risk of cataract formation by heightening the likelihood of lens touch and oxidative stress [1, 21]. As for RD, the high recurrence rate and the frequent need for tamponade likely explain the increased cataract risk in this group.

The main limitation of this study is its retrospective design, with many patients lost to follow-up. Some patients were referred to our center for surgery but continued their follow-up with their referring physician, leading to potential follow-up bias. To mitigate this, we excluded infants with follow-up periods shorter than six months and used the Kaplan-Meier statistical model to estimate the incidence of cataract. Additionally, as a tertiary referral center with a dedicated pediatric vitreoretinal department, we may have received more complex cases, particularly those involving silicone oil, potentially leading to an overestimation of cataract incidence. Finally, patients were included over a period of 20 years during which machines, instrumentations, technics, and surgeon’s experience have evolved which might affect the rate of cataract development. The strength of this study lies in its large patient cohort, inclusion of varied PPV etiologies, and extended follow-up period.

## Conclusions

In conclusion, our study revealed that approximately one-third of children develop cataracts following pediatric PPV, with key risk factors including the use of silicone oil tamponade, gas tamponade and the need for multiple surgeries. As such, early and long-term follow-up is crucial after PPV, particularly in young children, where cataract development could increase the risk of amblyopia.

## Data Availability

No datasets were generated or analysed during the current study.

## Abbreviations

CI: Confidence interval
OR: Odd ratio
PFV: Persistent fetal vasculature
PSC: Posterior subcapsular cataract
PP: Posterior pole
PPV: Pars plana vitrectomy
RD: Retinal detachment
ROP: Retinopathy of prematurity
SD: Standard deviation
SE: Standard error
VH: Vitreous hemorrhage
